# Complexed Crystal Structure of *Saccharomyces cerevisiae* Dihydroorotase with Inhibitor 5-Fluoroorotate Reveals a New Binding Mode

**DOI:** 10.1155/2021/2572844

**Published:** 2021-09-30

**Authors:** Hong-Hsiang Guan, Yen-Hua Huang, En-Shyh Lin, Chun-Jung Chen, Cheng-Yang Huang

**Affiliations:** ^1^Life Science Group, Scientific Research Division, National Synchrotron Radiation Research Center, Hsinchu, Taiwan; ^2^School of Biomedical Sciences, Chung Shan Medical University, No. 110, Sec. 1, Chien-Kuo N. Road, Taichung City, Taiwan; ^3^Department of Beauty Science, National Taichung University of Science and Technology, No. 193, Sec. 1, San-Min Road, Taichung City, Taiwan; ^4^Department of Biotechnology and Bioindustry Sciences, National Cheng Kung University, Tainan City, Taiwan; ^5^Department of Physics, National Tsing Hua University, Hsinchu, Taiwan; ^6^Department of Biological Science and Technology, National Chiao Tung University, Hsinchu, Taiwan; ^7^Department of Medical Research, Chung Shan Medical University Hospital, No. 110, Sec. 1, Chien-Kuo N. Road, Taichung City, Taiwan

## Abstract

Dihydroorotase (DHOase) possesses a binuclear metal center in which two Zn ions are bridged by a posttranslationally carbamylated lysine. DHOase catalyzes the reversible cyclization of *N*-carbamoyl aspartate (CA-asp) to dihydroorotate (DHO) in the third step of the pathway for the biosynthesis of pyrimidine nucleotides and is an attractive target for potential anticancer and antimalarial chemotherapy. Crystal structures of ligand-bound DHOase show that the flexible loop extends toward the active site when CA-asp is bound (loop-in mode) or moves away from the active site, facilitating the product DHO release (loop-out mode). DHOase binds the product-like inhibitor 5-fluoroorotate (5-FOA) in a similar mode to DHO. In the present study, we report the crystal structure of DHOase from *Saccharomyces cerevisiae* (ScDHOase) complexed with 5-FOA at 2.5 Å resolution (PDB entry 7CA0). ScDHOase shares structural similarity with *Escherichia coli* DHOase (EcDHOase). However, our complexed structure revealed that ScDHOase bound 5-FOA differently from EcDHOase. 5-FOA ligated the Zn atoms in the active site of ScDHOase. In addition, 5-FOA bound to ScDHOase through the loop-in mode. We also characterized the binding of 5-FOA to ScDHOase by using the site-directed mutagenesis and fluorescence quenching method. Based on these lines of molecular evidence, we discussed whether these different binding modes are species- or crystallography-dependent.

## 1. Introduction

**Figure 1:**
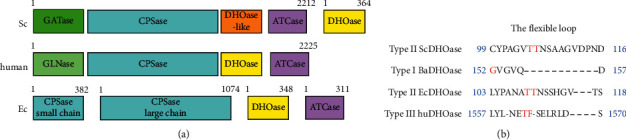
Comparison of DHOases. (a) The gene products for the first three reactions of pyrimidine biosynthesis are different among species. The higher eukaryotic human CAD consists of DHOase, CPSase, and ATCase domains fused covalently. Bacterial DHOase, CPSase, and ATCase function separately. However, CPSase and ATCase activities in *S. cerevisiae* are present in a single bifunctional protein, Ura2. Ura2 is a CAD-like polypeptide that contains a defective DHOase-like domain. (b) Sequence alignment of the flexible loop. The amino acids that are involved in catalysis are in red. The sequence composition and the length of these flexible loops are significantly distinct.

Dihydroorotase (DHOase) is a zinc metalloenzyme that catalyzes the reversible cyclization of *N*-carbamoyl aspartate (CA-asp) to dihydroorotate (DHO) in the third step of the pathway for the biosynthesis of pyrimidine nucleotides [1, 2]. The pharmacological inhibition of this pathway may provide an approach to targeting cancer cells, malarial parasites, and pathogens undergoing rapid growth [1–4]. In mammals, the activity of DHOase is found in a trifunctional enzyme, CAD, which also has activities of carbamoyl phosphate synthetase (CPSase) and aspartate transcarbamoylase (ATCase) [5]. However, significant variations are found in different species (Figure 1(a)). In fungi, CPSase and ATCase are present in a single bifunctional protein, Ura2, which is a CAD-like polypeptide that contains a defective DHOase-like domain [6]. In most prokaryotic organisms, CPSase, ATCase, and DHOase are expressed separately and function-independently [7]. *Pseudomonas aeruginosa* ATCase noncovalently associates with an inactive DHOase-like polypeptide for the ATCase activity [8]. *Aquifex aeolicus* DHOase (AaDHOase) is active only when complexed with AaATCase [9]. Thus, establishing the precise differences in DHOase among species is of considerable interest.

On the basis of known amino acid sequences and phylogenetic analyses, two major groups of DHOases are classified [10]. These two types of DHOases share a low level of protein sequence identity (less than 20%). AaDHOase, *Bacillus anthracis* DHOase (BaDHOase), and the DHOase domain (huDHOase) of human CAD are type I DHOases (about 45 kDa), which are evolutionarily ancient and larger than their type II counterparts (about 38 kDa), such as those from eubacteria, fungi, and plants. The recent structural analysis indicates that huDHOase should be reclassified as the type III DHOase [11] due to unique properties.

The type II DHOase from *Escherichia coli* (EcDHOase) was the first for which the structure was determined [12]. The complexed crystal structure showed that the substrate CA-asp and the product DHO were found at different active sites [12]. Further structural work indicated that a flexible loop extended toward the active site when CA-asp was bound (loop-in mode) or moved away from the active site, facilitating the product DHO release (loop-out mode) [13]. The mutagenesis study identified the importance of two Thr residues (T109 and T110) on the flexible loop in catalysis [14]. However, the sequence composition and the length of this loop (Figure 1(b)) in BaDHOase and huDHOase are significantly distinct [15]. In addition, the huDHOase chimera bearing the EcDHOase flexible loop is inactive, suggesting different catalytic specificities among species [16]. Thus, this loop as a lid within the active site of DHOase should be the prime target for selective inhibitor design.

5-Fluoroorotate (5-FOA) is a potent product-like inhibitor of DHOase from the malaria parasite *Plasmodium falciparum* [17]. The purified EcDHOase was inhibited by 5-FOA with *K*_*i*_ value of 31.8 *μ*M [13]. The binding modes of EcDHOase [13] and huDHOase [11] to 5-FOA were established using the structural information. In these complex structures, DHOase binds 5-FOA via the loop-out mode; namely, the flexible loops are not involved in the binding of 5-FOA. In the present study, we report the crystal structure of DHOase from *Saccharomyces cerevisiae* (ScDHOase) complexed with 5-FOA at 2.5 Å resolution (PDB entry 7CA0). ScDHOase shares structural similarity with EcDHOase. Given the same type of enzyme, one might conclude that the 5-FOA binding mode of ScDHOase must be similar to that of EcDHOase. However, we found that their 5-FOA binding modes were very different. We also characterized the binding of 5-FOA to ScDHOase by using the fluorescence quenching and mutational analysis.

## 2. Materials and Methods

### 2.1. Protein Expression and Purification

ScDHOase was purified using the protocol described previously [18, 19]. Briefly, *E. coli* BL21 (DE3) cells were transformed with the expression vector pET21b-ScDHOase, and the overexpression of the expression plasmids was induced by incubating with 1 mM isopropyl thiogalactopyranoside. The protein was purified from the soluble supernatant by using the Ni^2+^-affinity chromatography (HiTrap HP; GE Healthcare Bio-Sciences), eluted with Buffer A (20 mM Tris-HCl, 250 mM imidazole, and 0.5 M NaCl, pH 7.9), and dialyzed against a dialysis buffer (20 mM Tris-HCl and 0.1 M NaCl, pH 7.9). The protein purity remained at >97% as determined using SDS-PAGE (Mini-PROTEAN Tetra System; Bio-Rad, CA, USA).

### 2.2. Site-Directed Mutagenesis

The ScDHOase mutants were generated according to the QuikChange Site-Directed Mutagenesis Kit protocol (Stratagene; La Jolla, CA, USA), by using the wild-type plasmid pET21b-ScDHOase as a template. The presence of the mutation was verified by DNA sequencing in each construct. The recombinant mutant proteins were purified using the protocol for the wild-type ScDHOase by Ni^2+^-affinity chromatography.

### 2.3. Crystallization Experiment

Before crystallization, the purified ScDHOase was concentrated to 11 mg/mL. The crystals of ScDHOase complexed with 5-FOA were grown at room temperature through the hanging drop vapor diffusion in 16% PEG 4000 and 100 mM imidazole-malate, pH 6.8. The crystals of ScDHOase were validated in the beamline TLS 15A1 of the National Synchrotron Radiation Research Center (NSRRC; Hsinchu, Taiwan).

### 2.4. X-Ray Diffraction Data and Structure Determination

The native and the Zn-anomalous data were collected at beamline BL44XU at SPring-8 (Harima, Japan) with MX300-HE CCD detector and at beamline TPS 05A1 at the NSRRC (Hsinchu, Taiwan) with MX300-HS CCD detector. Datasets were indexed, integrated, and scaled by HKL-2000 [20] and XDS [21]. The initial phase, density modification, and model building were performed using the AutoSol program [22] in the PHENIX. The iterative model building and the structure refinement were performed using Refmac in the CCP4 software suite [23] and Phenix refine in the PHENIX software suite [24]. The initial phases of ScDHOase complexed with 5-FOA were determined through the molecular replacement software Phaser-MR [25] by using the monomeric ScDHOase derived from ScDHOase-malate complex [18] as the search model. After 12 cycles of model refinements, the best model of ScDHOase was utilized to calculate the real-space averaged OMIT |*F*_*o*_| − |*F*_*c*_| map using mapmask and maprot in the CCP4 software suite [23]. Based on the real-space averaged OMIT |*F*_*o*_| − |*F*_*c*_| map, the 5-FOA position was determined. Coot was used for manual model corrections and density fit analyses [26, 27]. The density fit value of 5-FOA is 0.95 using density fit analysis in Coot. The correctness of the stereochemistry of the models was verified using MolProbity [28]. Atomic coordinates and related structure factors were deposited in the PDB with accession code 7CA0.

### 2.5. Determination of the Dissociation Constant (*K*_*d*_)

The *K*_*d*_ value of the purified ScDHOase was determined using the fluorescence quenching method as previously described for dihydropyrimidinase (DHPase) [29–31]. Briefly, an aliquot of the compound was added to the solution containing ScDHOase (1 *μ*M) and 50 mM HEPES at pH 7.0. The decrease in the intrinsic fluorescence of ScDHOase was measured at 324 nm upon excitation at 280 nm and 25°C with a spectrofluorimeter (Hitachi F-2700; Hitachi High-Technologies, Japan). *K*_d_ was obtained using the following equation: Δ*F* = Δ*F*_max_ − *K*_*d*_ (Δ*F*/[compound]).

## 3. Results and Discussion

The DHOase activity is found in all organisms for the biosynthesis of pyrimidine nucleotides, but phylogenetic and structural analyses reveal at least three different DHOase forms [2, 11]. In bacteria and yeast, DHOase is monofunctional and belongs to the type II enzyme. As a eukaryotic DHOase, ScDHOase may be an evolutionary link between the Gram-negative bacterial DHOase (type II) and the higher eukaryotic DHOase domain of CAD (type III). Thus, the important differences between the prokaryotic EcDHOase and the eukaryotic ScDHOase are worth investigating.

### 3.1. Crystallization

We attempted to crystallize the ScDHOase-5-FOA complex by crystallization screening, but no crystal was formed. ScDHOase formed crystals only in the presence of malate [18]. Thus, we incubated 5-FOA with the crystal of the ScDHOase-malate complex and obtained the crystal of the ScDHOase-5-FOA complex successfully. The crystals of the ScDHOase complex belonged to space group *P*2_1_ with cell dimensions of *a* = 85.47, *b* = 88.59, and *c* = 103.57 Å. The crystal structure of ScDHOase complexed with 5-FOA was solved at 2.5 Å resolution (Table 1).

### 3.2. Crystal Structure of ScDHOase Complexed with 5-FOA

**Figure 2:**
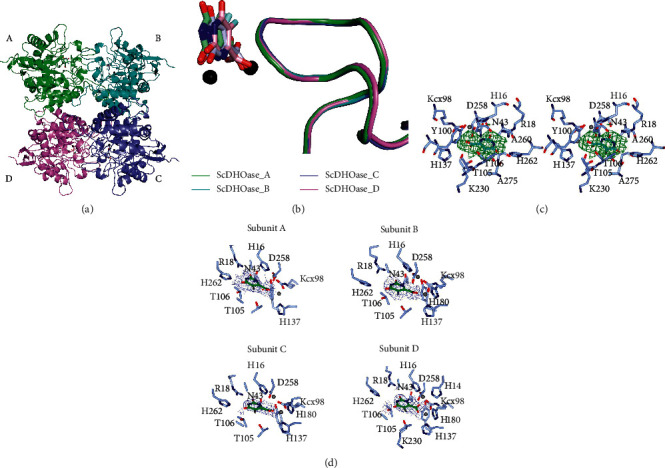
Structure of ScDHOase complexed with 5-FOA. (a) Ribbon diagram of the ScDHOase-5-FOA complex tetramer. Each monomer is color-coded. Two zinc ions in the active site are presented as black spheres. (b) Structural comparison of the active sites in ScDHOase tetramer. The superimposed structures of these 5-FOA-bound states revealed that the flexible loop is in loop-in conformation in each subunit. (c) Stereo view of the real-space averaged OMIT |*F*_*o*_| − |*F*_*c*_| map (green mesh, contoured at 3*σ*) of 5-FOA bound to ScDHOase. The bound 5-FOA (cyan) and ScDHOase (light blue) are shown as sticks. The carboxylate group of 5-FOA ligates the Zn atoms (gray spheres). (d) The composite OMIT map (blue mesh, contoured at 1*σ*) of 5-FOA bound to ScDHOase. The bound 5-FOA (green) and ScDHOase (light blue) are shown as sticks. Two zinc atoms are shown as gray spheres.

An asymmetric unit of the crystal contained four crystallography-independent ScDHOase monomers (Figure 2(a)). The global architecture of each ScDHOase monomer showed a TIM-barrel structure and consisted of 15 *α*-helices, 12 *β*-sheets, 2 Zn ions, and 1 5-FOA molecule. The Lys residue (K98) remained carbamylated regardless of 5-FOA binding. The dimetal center (Zn*α*/Zn*β*) in ScDHOase containing 4 His (i.e., H14, H16, H137, and H180), 1 Asp (i.e., D258), and 1 carbamylated Lys (i.e., Kcx98) was still self-assembled. The structure revealed a long flexible loop in each subunit which extended toward the active site when 5-FOA was bound (Figure 2(b)). The occupancy refinement was performed using the PHENIX.refine software [24]. The occupancy of 5-FOA in each subunit is 0.74–0.78. Possibly, the partial occupancy of 5-FOA resulted from the replacement or disturbance of malate in the mother liquid [18]. The two Thr residues, Thr109 and Thr110 in EcDHOase, important for stabilizing the transition state but not interacting with 5-FOA [13], were also conserved in ScDHOase (Thr105 and Thr106). However, these two Thr residues did interact with 5-FOA revealed by our complex structure.

### 3.3. 5-FOA Binding Mode

The binding modes of EcDHOase [13] and huDHOase [11] to 5-FOA were well established using the structural information. In these structures, 5-FOA bound to the active site in a similar mode to DHO. However, our complexed structure revealed that ScDHOase bound 5-FOA (Figures 2(c) and 2(d)) differently from EcDHOase and huDHOase. To confirm the different binding mode, we compared their density fit values of the bound 5-FOA for ScDHOase using Coot. The binding mode of 5-FOA observed from EcDHOase and huDHOase was refined using the rigid body real-space refinement in Coot to the averaged omit map for ScDHOase. The density fit value of the bound 5-FOA in ScDHOase to the averaged omit map is 0.95. When using the posture of 5-FOA from EcDHOase and huDHOase, the density fit value of the bound 5-FOA in ScDHOase is 0.81. Based on these results, we ruled out the possibility that the 5-FOA binding mode of ScDHOase must be similar to that of EcDHOase and huDHOase.

To strengthen the conclusion that ScDHOase bound 5-FOA differently from EcDHOase and huDHOase, we also checked the averaged RMSZs (indicators of ligand geometry) of 5-FOA in our complexed structure of ScDHOase (PDB entry 7CA0; 1.58 of bond lengths and 2.02 of bond angles) and compared with the reported 5-FOA structures bound in huDHOase (PDB entry 4C6M; 4.12 of bond lengths and 6.11 of bond angles) and in EcDHOase (PDB entry 2EG8, 3.0 of bond lengths and 4.58 of bond angles). Based on these values, the proposed 5-FOA binding mode of ScDHOase through the complex structure is reasonable and evident.

**Figure 3:**
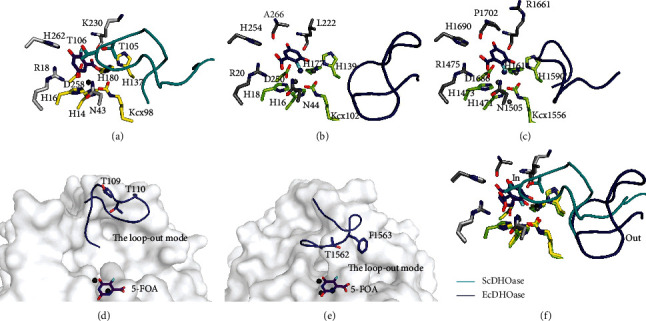
New binding mode of 5-FOA. (a) The active site of ScDHOase with 5-FOA. (b) The active site of EcDHOase with 5-FOA (PDB entry 2EG8). The binding mode of 5-FOA to the active site is very similar to that of DHO. (c) The active site of huDHOase with 5-FOA (PDB entry 4C6M). By comparison, the bound 5-FOA by ScDHOase adopted the reverse orientation as compared to that in the active site of EcDHOase and huDHOase. (d) The loop-out binding mode of EcDHOase. (e) The loop-out binding mode of huDHOase. The flexible loop in EcDHOase and huDHOase is not involved in the binding of 5-FOA. (f) Superposition of the ScDHOase and EcDHOase complexes. Despite having a similar active site, the 5-FOA binding pose and the conformation of the catalytic loop of ScDHOase differ from those of EcDHOase.

For ScDHOase, the carboxylate group of 5-FOA ligated the Zn atoms (Figure 3(a)) rather than interacting with the positively charged side chain of Arg18 as that in EcDHOase (Figure 3(b)) and huDHOase (Figure 3(c)). The bound 5-FOA by ScDHOase adopted the reverse orientation compared with those by EcDHOase and huDHOase. In addition, all flexible loops in each subunit of ScDHOase were involved in the binding of 5-FOA (Figure 2(b)). The two Thr residues (Thr105 and Thr106) in ScDHOase played a crucial role in binding. For EcDHOase (Figure 3(d)) and huDHOase (Figure 3(e)), the flexible loop is not involved in the binding of 5-FOA. Despite having a similar active site, the 5-FOA binding pose and the conformation of the catalytic loop of ScDHOase for 5-FOA binding (via the loop-in mode) differed from those of EcDHOase (Figure 3(f)) and huDHOase (via the loop-out mode). We concluded that the 5-FOA binding and the inhibition mechanism of ScDHOase were different from those of EcDHOase and huDHOase.

**Figure 4:**
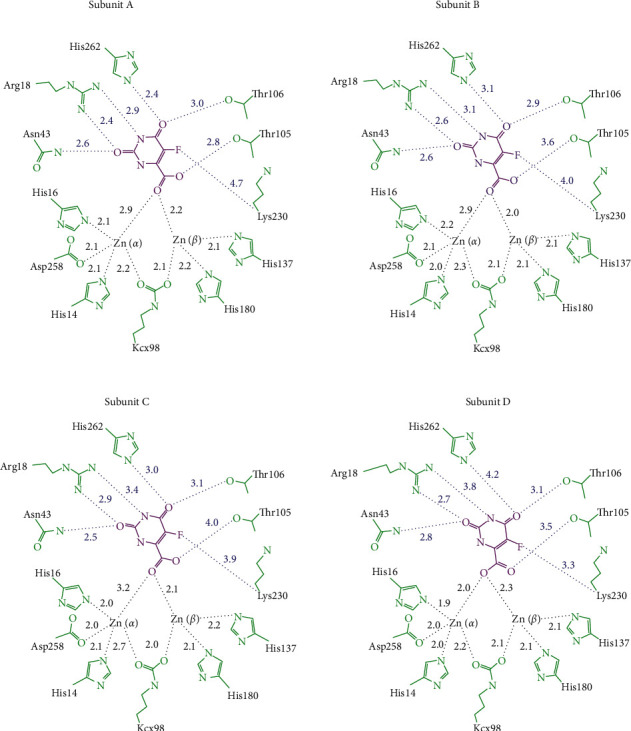
5-FOA binding mode of ScDHOase. Arg18, Asn43, His262, Thr105, Thr106, and Lys230 of ScDHOase were involved in the 5-FOA binding.

According to our structure, Arg18, Asn43, His262, Thr105, Thr106, and Lys230 of ScDHOase were involved in the 5-FOA binding (Figure 4). The 5-FOA binding mode for ScDHOase was somehow similar to the binding mode of EcDHOase to 2-oxo-1,2,3,6-tetrahydropyrimidine-4,6-dicarboxylic acid (HDDP) [13]. EcDHOase bound HDDP via the loop-in mode. Similarly, the bound HDDP in EcDHOase utilized its carboxylate group to interact with Zn atoms and stabilize the flexible loop. Like the ScDHOase-5-FOA complex, Thr109 and Thr110 in EcDHOase played a crucial role in the binding of HDDP.

### 3.4. Binding and Mutational Analysis

**Figure 5:**
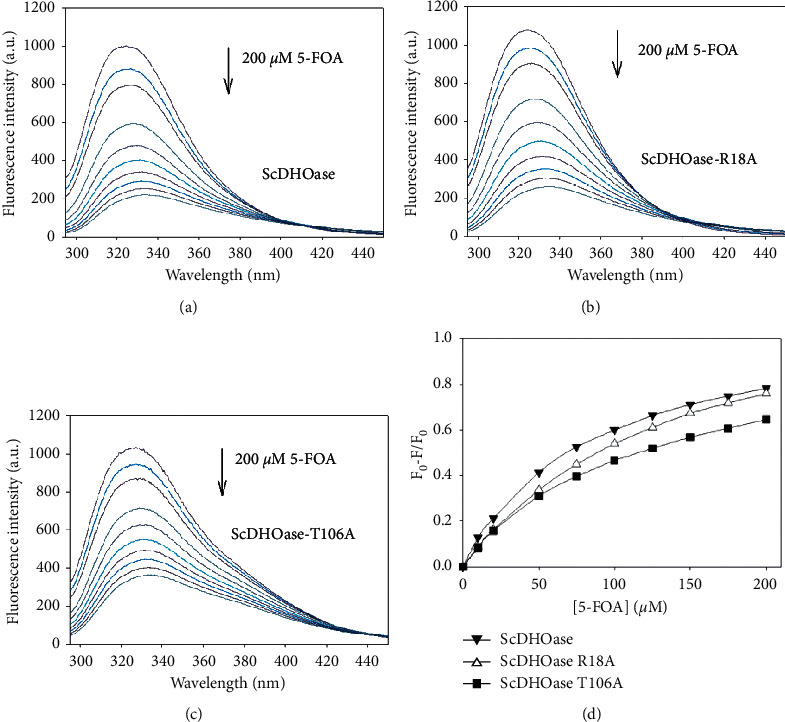
Fluorescence titration of ScDHOase with 5-FOA. (a) The fluorescence emission spectra of ScDHOase with 5-FOA of different concentrations (0–200 *μ*M; 0, 10, 20, 50, 75, 100, 125, 150, 175, and 200 *μ*M). The decrease in intrinsic fluorescence of protein was measured at 324 nm upon excitation at 280 nm with a spectrofluorimeter. The fluorescence intensity emission spectra of ScDHOase significantly quenched with 5-FOA. (b) The fluorescence emission spectra of ScDHOase-R18A with 5-FOA of different concentrations (0–200 *μ*M). (c) The fluorescence emission spectra of ScDHOase-T106A with 5-FOA of different concentrations (0–200 *μ*M). ScDHOase-R18A and ScDHOase-T106A individually displayed strong intrinsic fluorescence with a peak wavelength of 324.5 and 328 nm when excited at 280 nm. (d) An aliquot amount of 5-FOA was added to the enzyme solution for determining *K*_*d*_. *K*_*d*_ was obtained by the following equation: Δ*F* = Δ*F*_max_ − *K*_*d*_ (Δ*F*/[5-FOA]). Data points are an average of 2-3 determinations within 10% error.

Fluorescence quenching method was used for determining the dissociation constant (*K*_*d*_) of ScDHOase bound to 5-FOA (Figure 5(a)). *K*_*d*_ of the ScDHOase mutants R18A (Figure 5(b)) and T106A (Figure 5(c)) was also determined through the fluorescence quenching to confirm the strength of interaction of ScDHOase with 5-FOA. Quenching refers to the complex formation process that decreases the fluorescence intensity of the protein. ScDHOase displayed strong intrinsic fluorescence with a peak wavelength of 324 nm when excited at 280 nm (Figure 5(a)). When 5-FOA was titrated into the ScDHOase solution, the intrinsic fluorescence of the protein was progressively quenched. Upon the addition of 200 *μ*M 5-FOA, the intrinsic fluorescence of ScDHOase, ScDHOase-R18A, and ScDHOase-T106A was quenched by 78.2%, 75.9%, and 64.6%, respectively (Table 2). Adding 5-FOA resulted in a red shift in the ScDHOase emission wavelength (∼11.5 nm; *λ*_max_ from 324.0 to 335.5 nm). Adding 5-FOA also resulted in red shifts in the ScDHOase-R18A (∼10.0 nm; *λ*_max_ from 324.5 to 334.5 nm) and ScDHOase-T106A (∼5.5 nm; *λ*_max_ from 328.0 to 333.5 nm). The *λ*_em_ shift of ScDHOase-T106A (5.5 nm) produced by 5-FOA was significantly lower than that of ScDHOase (11.5 nm). These observations indicated that ScDHOase, ScDHOase-R18A, and ScDHOase-T106A could form a stable complex with 5-FOA, respectively; however, the binding affinities for these ScDHOases were different. As determined through the titration curves (Figure 5(d)), the *K*_*d*_ values of ScDHOase, ScDHOase-R18A, and ScDHOase-T106A bound to 5-FOA were 83.8 ± 1.5, 143.6 ± 2.1, and 114.8 ± 3.7 *μ*M, respectively. We also compared the binding affinities of ScDHOase to the anticancer drugs 5-fluorouracil (5-FU) and 5-aminouracil (5-AU). Based on the *K*_*d*_ values, the strength of complex formation followed the following order: ScDHOase-5-FOA > ScDHOase-R18A-5-FOA > ScDHOase-T106A-5-FOA > ScDHOase-5-FU > ScDHOase-5-AU [18]. Thus, ScDHOase preferred the binding of 5-FOA (Table 2) over 5-FU and 5-AU [18].

The decrease in the intrinsic fluorescence of ScDHOase was measured with a spectrofluorimeter (Hitachi F-2700; Hitachi High-Technologies, Japan). *K*_*d*_ was obtained using the following equation: Δ*F* = Δ*F*_max_ − *K*_*d*_ (Δ*F*/[5-FOA]).

### 3.5. Binding of 5-FOA via Loop-In Mode

Despite the evolutionary divergence (Figure 1), an important flexible loop as a lid within the active site of DHOase for catalysis and substrate binding is conserved from *E. coli* [13] to humans [11]. 5-FOA, a product-like inhibitor, binds to the active site of EcDHOase in a similar manner to DHO via the loop-out binding mode (Figures 2 and 3); that is, the loop does not interact with the ligand or with the rest of the active site [13]. Despite a very similar active site, ScDHOase bound 5-FOA by using different mechanism. Through the loop-in mode, the bound 5-FOA by ScDHOase adopted the reverse orientation, as compared with that by EcDHOase. We also observed that ScDHOase bound 5-AU, 5-FU, and malate via the loop-in mode [18]. To date, we have not found the loop-out mode of ScDHOase to bind ligand. Whether ScDHOase can bind ligand via the loop-out conformation is still unknown. Given that the flexible loop in ScDHOase is the longest among these DHOases (Figure 1(b)), they may be somehow different in their binding mechanisms (Figure 3). Perhaps, the conformational change of this loop in ScDHOase is not necessary due to the steric hindrance. Whether these different binding modes are species- or crystallography-dependent should be elucidated experimentally.

The loop-in binding mode is also found in DHPase [29, 32, 33] and allantoinase (ALLase) [34]. DHOase [1], DHPase [35–37], and ALLase [38, 39] are members of the cyclic amidohydrolase family [32, 40]. These metal-dependent enzymes catalyze the hydrolysis of the cyclic amide bond of each substrate in either 5- or 6-membered rings in the metabolism of purines and pyrimidines [1, 32]. The conserved Tyr residue located within a dynamic loop in DHPase [30, 33] plays an essential role in the stabilization of the tetrahedral transition state during hydrolysis of the substrate, collapse of the transition state, formation of a product, and release of the product. Thus, the dynamic loop in these cyclic amidohydrolases could be a suitable drug target for inhibitor design [30, 41]. Structural analyses are still needed to decipher the architecture and the function of different DHOases.

## 4. Conclusion

The complexed crystal structure of ScDHOase with inhibitor 5-FOA determined at 2.5 Å resolution revealed a new binding mode. Although ScDHOase shares structural similarity with EcDHOase, they appear to bind 5-FOA differently. We also characterized the binding of 5-FOA to ScDHOase by using the fluorescence quenching and mutational analysis. Through the loop-in mode, the conserved Thr residue located within a flexible loop in ScDHOase was crucial for binding of 5-FOA. Structure-function analyses indicated that the inherent difference in the flexible loop among DHOase species may be a determinant of the 5-FOA binding mode. Further research can directly focus on determining why DHOases need to evolve the different flexible loops for catalysis during evolution.

## Figures and Tables

**Table 1:** Data collection and refinement statistics.

Data collection	
Crystal	5-FOA-ScDHOase
Wavelength (Å)	0.9 Å
Resolution (Å)	44.57–2.50 (2.589–2.5)
Space group	*P*2_1_
Cell dimension *a*, *b*, *c* (Å)/*β* (°)	85.47, 88.59, 103.57/95.3
Redundancy	2.0 (2.0)
Completeness (%)	98.05 (99.81)
<*I*/*σ_I_*>	8.2 (1.2)
CC_1/2_	0.997 (0.80)

Refinement	
Resolution (Å)	44.57–2.50
No. of reflections	52420
*R*_work_/*R*_free_	0.205/0.248
No. of atoms	
Ligands	56
Macromolecules	11376
Zinc	8
Water	186
Protein residues	1456

r.m.s deviations	
Bond lengths (Å)	0.010
Bond angles (°)	1.29

Ramachandran plot	
Favored (%)	96.45
Allowed (%)	3.55
Outliers (%)	0
PDB entry	7CA0

Values in parentheses are for the highest resolution shell. CC_1/2_ is the percentage of correlation between intensities of random half datasets.

**Table 2:** Binding parameters of ScDHOase to 5-FOA.

DHOase	*λ*_max_ (nm)	*λ*_em_ shift (nm)	Quenching (%)	*K*_*d*_ value (*μ*M)
ScDHOase	From 324 to 335.5	11.5	78.2	83.8 ± 1.5
ScDHOase-R18A	From 324.5 to 334.5	10.0	75.9	143.6 ± 2.1
ScDHOase-T106A	From 328 to 333.5	5.5	64.6	114.8 ± 3.7

## Data Availability

Atomic coordinates and related structure factors were deposited in the PDB with accession code 7CA0. All the data used to support the findings of this study are available from the corresponding author upon request.

## References

[1] del Caño-Ochoa F., Moreno-Morcillo M., Ramón-Maiques S. (2019). CAD, a multienzymatic protein at the head of de novo pyrimidine biosynthesis. *Subcellular Biochemistry*.

[2] Evans D. R., Guy H. I. (2004). Mammalian pyrimidine biosynthesis: fresh insights into an ancient pathway. *Journal of Biological Chemistry*.

[3] Samant S., Lee H., Ghassemi M. (2008). Nucleotide biosynthesis is critical for growth of bacteria in human blood. *PLoS Pathogens*.

[4] Lipowska J., Miks C. D., Kwon K. (2019). Pyrimidine biosynthesis in pathogens—structures and analysis of dihydroorotases from *Yersinia pestis* and *Vibrio cholerae*. *International Journal of Biological Macromolecules*.

[5] Lee L., Kelly R. E., Pastra-Landis S. C., Evans D. R. (1985). Oligomeric structure of the multifunctional protein CAD that initiates pyrimidine biosynthesis in mammalian cells. *Proceedings of the National Academy of Sciences*.

[6] Souciet J. L., Nagy M., Le Gouar M., Lacroute F., Potier S. (1989). Organization of the yeast URA2 gene: identification of a defective dihydroorotase-like domain in the multifunctional carbamoylphosphate synthetase-aspartate transcarbamylase complex. *Gene*.

[7] Washabaugh M. W., Collins K. D. (1984). Dihydroorotase from *Escherichia coli*. purification and characterization. *Journal of Biological Chemistry*.

[8] Patel C., Vaishnav A., Edwards B. F. P., Evans D. R. (2020). Characterization and assembly of the *Pseudomonas aeruginosa* aspartate transcarbamoylase-pseudo dihydroorotase complex. *PLoS One*.

[9] Ahuja A., Purcarea C., Ebert R., Sadecki S., Guy H. I., Evans D. R. (2004). *Aquifex aeolicus* dihydroorotase: association with aspartate transcarbamoylase switches on catalytic activity. *Journal of Biological Chemistry*.

[10] Simmer J. P., Kelly R. E., Rinker A. G. (1990). Mammalian dihydroorotase: nucleotide sequence, peptide sequences, and evolution of the dihydroorotase domain of the multifunctional protein CAD. *Proceedings of the National Academy of Sciences*.

[11] Grande-García A., Lallous N., Díaz-Tejada C., Ramón-Maiques S. (2014). Structure, functional characterization, and evolution of the dihydroorotase domain of human CAD. *Structure*.

[12] Thoden J. B., Phillips G. N., Neal T. M., Raushel F. M., Holden H. M. (2001). Molecular structure of dihydroorotase: a paradigm for catalysis through the use of a binuclear metal center. *Biochemistry*.

[13] Lee M., Chan C. W., Graham S. C., Christopherson R. I., Guss J. M., Maher M. J. (2007). Structures of ligand-free and inhibitor complexes of dihydroorotase from *Escherichia coli*: implications for loop movement in inhibitor design. *Journal of Molecular Biology*.

[14] Lee M., Maher M. J., Christopherson R. I., Guss J. M. (2007). Kinetic and structural analysis of mutant *Escherichia coli* dihydroorotases: a flexible loop stabilizes the transition state,. *Biochemistry*.

[15] Rice A. J., Lei H., Santarsiero B. D., Lee H., Johnson M. E. (2016). Ca-asp bound X-ray structure and inhibition of *Bacillus anthracis* dihydroorotase (DHOase). *Bioorganic & Medicinal Chemistry*.

[16] del Caño-Ochoa F., Grande-García A., Reverte-López M., D’Abramo M., Ramón-Maiques S. (2018). Characterization of the catalytic flexible loop in the dihydroorotase domain of the human multi-enzymatic protein CAD. *Journal of Biological Chemistry*.

[17] Rathod P. K., Khatri A., Hubbert T., Milhous W. K. (1989). Selective activity of 5-fluoroorotic acid against *Plasmodium falciparum* in vitro. *Antimicrobial Agents and Chemotherapy*.

[18] Guan H.-H., Huang Y.-H., Lin E.-S., Chen C.-J., Huang C.-Y. (2021). Structural basis for the interaction modes of dihydroorotase with the anticancer drugs 5-fluorouracil and 5-aminouracil. *Biochemical and Biophysical Research Communications*.

[19] Huang Y.-H., Huang C.-Y. (2015). Creation of a putative third metal binding site in type II dihydroorotases significantly enhances enzyme activity. *Protein & Peptide Letters*.

[20] Otwinowski Z., Minor W. (1997). [20] processing of X-ray diffraction data collected in oscillation mode. *Methods in Enzymology*.

[21] Kabsch W. (2010). XDS. *Acta Crystallographica Section D Biological Crystallography*.

[22] Terwilliger T. C., Adams P. D., Read R. J. (2009). Decision-making in structure solution using Bayesian estimates of map quality: the PHENIX AutoSolwizard. *Acta Crystallographica Section D Biological Crystallography*.

[23] Lebedev A. A., Young P., Isupov M. N., Moroz O. V., Vagin A. A., Murshudov G. N. (2012). JLigand: a graphical tool for the CCP4 template-restraint library. *Acta Crystallographica Section D Biological Crystallography*.

[24] Terwilliger T. C., Grosse-Kunstleve R. W., Afonine P. V. (2008). Iterative model building, structure refinement and density modification with the PHENIX AutoBuild wizard. *Acta Crystallographica Section D Biological Crystallography*.

[25] Winn M. D., Ballard C. C., Cowtan K. D. (2011). Overview of the CCP4 suite and current developments. *Acta Crystallographica Section D Biological Crystallography*.

[26] Emsley P., Cowtan K. (2004). Coot: model-building tools for molecular graphics. *Acta Crystallographica Section D Biological Crystallography*.

[27] Emsley P., Lohkamp B., Scott W. G., Cowtan K. (2010). Features and development of coot. *Acta Crystallographica Section D Biological Crystallography*.

[28] Chen V. B., Arendall W. B., Headd J. J. (2010). MolProbity: all-atom structure validation for macromolecular crystallography. *Acta Crystallographica Section D Biological Crystallography*.

[29] Huang Y.-H., Lien Y., Chen J.-H., Lin E.-S., Huang C.-Y. (2020). Identification and characterization of dihydropyrimidinase inhibited by plumbagin isolated from *Nepenthes miranda* extract. *Biochimie*.

[30] Huang Y.-H., Ning Z.-J., Huang C.-Y. (2019). Crystal structure of dihydropyrimidinase in complex with anticancer drug 5-fluorouracil. *Biochemical and Biophysical Research Communications*.

[31] Huang C.-Y. (2015). Inhibition of a putative dihydropyrimidinase from *Pseudomonas aeruginosa* PAO1 by flavonoids and substrates of cyclic amidohydrolases. *PLoS One*.

[32] Huang C.-Y. (2020). Structure, catalytic mechanism, posttranslational lysine carbamylation, and inhibition of dihydropyrimidinases. *Advances in Protein Chemistry and Structural Biology*.

[33] Hsieh Y.-C., Chen M.-C., Hsu C.-C., Chan S. I., Yang Y.-S., Chen C.-J. (2013). Crystal structures of vertebrate dihydropyrimidinase and complexes from *Tetraodon nigroviridis* with lysine carbamylation: metal and structural requirements for post-translational modification and function. *Journal of Biological Chemistry*.

[34] Kim K., Kim M.-I., Chung J., Ahn J.-H., Rhee S. (2009). Crystal structure of metal-dependent allantoinase from *Escherichia coli*. *Journal of Molecular Biology*.

[35] Cheng J.-H., Huang Y.-H., Lin J.-J., Huang C.-Y. (2018). Crystal structures of monometallic dihydropyrimidinase and the human dihydroorotase domain K1556A mutant reveal no lysine carbamylation within the active site. *Biochemical and Biophysical Research Communications*.

[36] Cheng J. H., Huang C. C., Huang Y. H., Huang C. Y. (2018). Structural basis for pH-dependent oligomerization of dihydropyrimidinase from Pseudomonas aeruginosa PAO1. *Bioinorganic Chemistry and Applications*.

[37] Tzeng C.-T., Huang Y.-H., Huang C.-Y. (2016). Crystal structure of dihydropyrimidinase from *Pseudomonas aeruginosa* PAO1: insights into the molecular basis of formation of a dimer. *Biochemical and Biophysical Research Communications*.

[38] Peng W.-F., Huang C.-Y. (2014). Allantoinase and dihydroorotase binding and inhibition by flavonols and the substrates of cyclic amidohydrolases. *Biochimie*.

[39] Ho Y.-Y., Huang Y.-H., Huang C.-Y. (2013). Chemical rescue of the post-translationally carboxylated lysine mutant of allantoinase and dihydroorotase by metal ions and short-chain carboxylic acids. *Amino Acids*.

[40] Gerlt J. A., Babbitt P. C. (2001). Divergent evolution of enzymatic function: mechanistically diverse superfamilies and functionally distinct suprafamilies. *Annual Review of Biochemistry*.

[41] Guan H.-H., Huang Y.-H., Lin E.-S., Chen C.-J., Huang C.-Y. (2021). Plumbagin, a natural product with potent anticancer activities, binds to and inhibits dihydroorotase, a key enzyme in pyrimidine biosynthesis. *International Journal of Molecular Sciences*.

